# Genotyping of human rhinovirus in adult patients with acute respiratory infections identified predominant infections of genotype A21

**DOI:** 10.1038/srep41601

**Published:** 2017-01-27

**Authors:** Lili Ren, Donghong Yang, Xianwen Ren, Mingkun Li, Xinlin Mu, Qi Wang, Jie Cao, Ke Hu, Chunliang Yan, Hongwei Fan, Xiangxin Li, Yusheng Chen, Ruiqin Wang, Fucheng An, Shuchang An, Ming Luo, Ying Wang, Yan Xiao, Zichun Xiang, Yan Xiao, Li Li, Fang Huang, Qi Jin, Zhancheng Gao, Jianwei Wang

**Affiliations:** 1 MOH Key Laboratory of Systems Biology of Pathogens and Christophe Mérieux Laboratory, IPB, CAMS-Fondation Mérieux, Institute of Pathogen Biology (IPB), Chinese Academy of Medical Sciences (CAMS) & Peking Union Medical College, Beijing 100730, P. R. China; 2 Department of Respiratory and Critical Care Medicine, Peking University People’s Hospital, Beijing, 100044, P. R. China; 3 MOH Key Laboratory of Systems Biology of Pathogens, Institute of Pathogen Biology, Chinese Academy of Medical Sciences & Peking Union Medical College, Beijing, 100176, P. R. China; 4 Fondation Mérieux, Lyon 69365, France; 5 Department of Respiratory Medicine, The Second Affiliated Hospital of Dalian Medical University, Dalian, 116027, P. R. China; 6 Department of Respiratory Medicine, Tianjin Medical University General Hospital, Tianjin 300052, P. R. China; 7 Department of Respiratory Medicine, Renmin Hospital of Wuhan University, Wuhan, 430060, P. R. China; 8 Department of Respiratory & Critical Care Medicine, Beijing Aerospace General Hospital, Beijing, 100076, P. R. China; 9 Peking Union Medical College Hospital, Chinese Academy of Medical Sciences & Peking Union Medical College, Beijing, 100730, P. R. China; 10 Department of Respiratory Medicine, Beijing Changping Hospital, Beijing, 102200, P. R. China; 11 Department of Respiratory Medicine, Fujian Provincial Hospital, Fuzhou, 350001, P. R. China; 12 Department of Respiratory Medicine, The First Affiliated Hospital of Tsinghua University, Beijing, 100016, P. R. China; 13 Department of Respiratory Medicine, Mentougou District Hospital, Beijing, 102300, P. R. China; 14 Beijing Center for Disease Prevention and Control, No. 16, Hepingli Middle Avenue of Dongcheng district, Beijing, 100013, P. R. China; 15 Institute of Pathogen Biology, Chinese Academy of Medical Sciences & Peking Union Medical College, Beijing, 100176, P. R. China

## Abstract

Human rhinovirus (HRV) is an important causative agent of acute respiratory tract infections (ARTIs). The roles of specific HRV genotypes in patients suffering from ARTIs have not been well established. We recruited 147 adult inpatients with community-acquired pneumonia (CAP) and 291 adult outpatients with upper ARTIs (URTIs). Respiratory pathogens were screened via PCR assays. HRV was detected in 42 patients, with 35 species A, five B and two C. Seventeen genotypes were identified, and HRV-A21 ranked the highest (9/42, 21.4%). The HRV-A21-positive infections were detected in four patients with CAP and in five with URTIs, all without co-infections. The HRV-A21 genome sequenced in this study contained 12 novel coding polymorphisms in viral protein (VP) 1, VP2 EF loop, VP3 knob and 3D regions. The infections of HRV-A21 virus obtained in this study could not be neutralized by antiserum of HRV-A21 prototype strain (VR-1131), indicating remarkable antigenic variation. Metagenomic analysis showed the HRV-A21 reads were dominant in bronchoalveolar lavage fluid of the three HRV-A21-positive patients with severe CAP, in which two dead. Our results highlight an unexpected infection of genotype HRV-A21 in the clinic, indicating the necessity of precise genotyping and surveillance of HRVs to improve the clinical management of ARTIs.

Respiratory viruses are the major etiological agent of acute respiratory tract infections (ARTIs)^1^. The surveillance of respiratory viruses, especially in severe lower ARTI (LRTI) cases have been attributed to the identification of emerging and re-emerging respiratory viruses that have the potential to threaten global public health^2^,^3^. New viral pathogens, such as influenza A H1N1pdm, Middle East respiratory syndrome coronavirus (MERS-CoV), and avian influenza AH7N9 virus, are continually emerging^3^. Meanwhile, certain preexisting pathogens, which may have gone undetected before, have presented with new pathogenic features due to variation, leading to severe diseases and/or public health concerns. For instance, enterovirus (EV) D68, a rarely reported virus that has been spreading worldwide over recent years and caused a pandemic in the USA in 2014, is associated with severe pneumonia and even acute flaccid myelitis^4^. The precise identification of the related pathogens in the clinic is important for early intervention and preventing its potential to spread in the public^2^,^3^,^4^.

Human rhinovirus (HRV), a member of the enterovirus genus in the *Picornaviridae* family, is considered to be an important human respiratory pathogen^5^. HRVs are one of the most frequent causes of ARTIs, and they are the primary cause of asthma exacerbation and chronic obstructive pulmonary disease (COPD)^5^,^6^. HRV is also associated with severe pneumonia, particularly in infants and adults with underlying diseases or in immunocompromised patients^7^,^8^,^9^,^10^. A total of 167 HRV genotypes belonging to three species (A, B, and C) have been identified (http://www.picornaviridae.com/enterovirus/enterovirus.htm). However, most of the previous studies have only evaluated the etiologic role of HRVs in ARTIs at the species level, the roles of the specific genotypes in ARTIs have not been well established.

Here, we analyze the prevalence of HRV and its genotypes in ARTI patients and report predominant infection of genotype A21 (HRV-A21). We also describe the clinical characteristics of patients with HRV-A21 infections and the viral genomic traits of this virus.

## Results

### Genotyping of human rhinoviruses in ARTI patients

To identify the roles of HRV genotypes in adult ARTIs, we recruited 438 patients from January to December 2013, including 147 community-acquired pneumonia (CAP) inpatients (including 39 severe cases) and 291 Upper ARTI (URTI) outpatients. The CAP inpatients ranged from 18 to 92 years old, with a median age of 55.5 [Interquartile range (IQR) 35–67] years. The URTI outpatients ranged from 18 to 84 years old, with a median age of 30 (IQR 24–41) years.

A total of 169 (38.6%) patients tested positive for at least one respiratory pathogen via multiplex real-time PCR. Forty-two (9.6%) patients were positive for HRV, including 15 CAP and 27 URTI patients (Table 1). Co-detection was found in ten HRV-positive cases, three of which were CAP patients (co-detection with *Mycoplasma pneumoniae, Streptococcus pneumoniae*, or HCoV-229E, respectively). The other seven cases were URTI patients, four of whom were also infected with influenza viruses (IFVs), two with respiratory syncytial virus (RSV), and one with EV. HRV RNA was not detected in the sera of HRV-positive patients, as determined by RT-PCR. The HRV species for these patients were as follows: 35 HRV-A, 5 HRV-B, and 2 HRV-C. Among them, 29 HRV-A and 5 HRV-B were successfully genotyped.

In the CAP patients, there were four cases of HRV-A21 and two of A68. The rest of the genotypes were one case each of A2, A9, A13, A33, A53, B35, B48, B84, and C un-typed. Three of the four HRV-A21-positive CAP inpatients had serially collected respiratory samples, and all were positive for HRV-A21. In the URTI patients, there were five HRV-A21, three A61, two A98, and one case of each of the following genotypes: A2, A28, A53, A58, A60, A68, A96, B27, B84, and C un-typed. Seven samples of HRV-A in the URTI patients could not be genotyped precisely (Table 1). The detection rate of HRV-A21 was higher in the severe CAP patients (7.9%, n = 3) than in the non-severe CAP (0.9%, n = 1) or the URTI patients (1.7%, n = 5) (P = 0.047 by Fisher’s exact test). Taken together, these data show that prevalence of HRV-A21 correlates with a spectrum of illness from mild URTIs to severe CAP. Unexpectedly, HRV-A21 was most frequency (9 of 42, 21.4%) detected as the only pathogen present in an infection among the identified HRV genotypes. As only three genome sequences of HRV-A21 were available in GenBank and little was reported on its clinical manifestation, we focused on the characterization of A21 infections.

### Phylogeny, coding polymorphisms of HRV-A21 strains, and viral neutralizing activity

**Figure 1:**
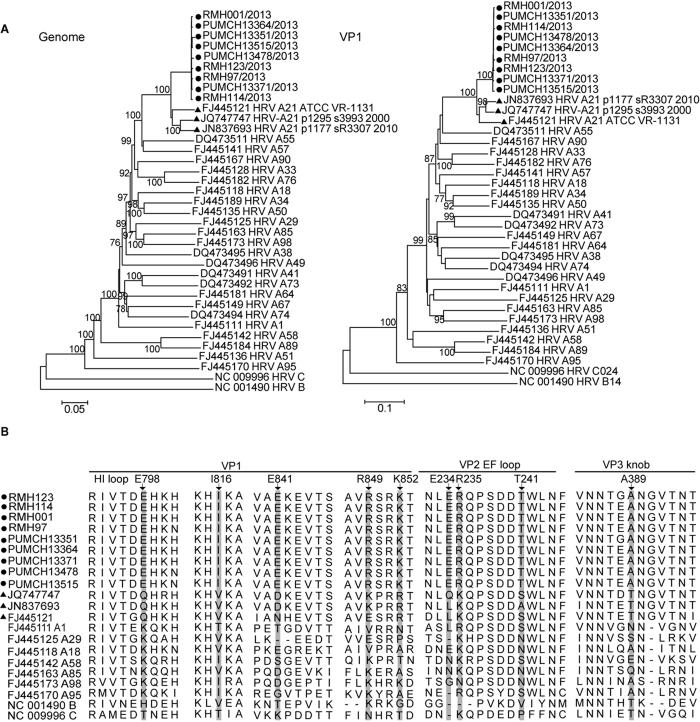
Phylogenic analysis and coding polymorphisms of HRV-A21. (**A**) The strains identified in this study (dark circles), reference sequences (triangles), and outgroup sequences were analyzed, and phylogenic trees of the genomes and viral protein gene (VP) 1 sequences were constructed using the neighbor-joining method (Kimura’s two-parameter) with 1,000 bootstrap values. (**B**) Coding polymorphisms in VP1, the VP2 EF loop, and the VP3 knob regions.

Complete genomic sequences were obtained from all nine HRV-A21-positive cases. Phylogenetic analysis showed that the HRV-A21 strains identified in this study were closely related to the three available reference sequences from GenBank, including the prototype strain (ATCC VR-1131; FJ445121) and two strains identified in the USA in 2000 (p1295_s3993_2000; JQ747747) and 2010 (p1177_sR3307_2010; JN837693) (Fig. 1A), but they formed a distinct clade in the phylogenetic trees of complete viral genome and VP1 gene), indicating the presence of variants of the newly isolated HRV-A21 strains. Multiple alignment analyses among the HRV-A21 strains in this study showed that the nucleotide (nt) identities of the genomes were 99.0–99.7%, whereas the amino acid (aa) sequence identities of VP1 were 98.6–100.0%. Using the RMH001/2013 strain as a representative, the nt and aa sequences of VP1 compared to the reference sequences were 90.4–92.1% and 94.7–96.5%, respectively (Table 2).

The genetic diversity of the newly identified HRV-A21 was further characterized by aa alignments. A total of 12 specific aa substitutions in the polyproteins of the HRV-A21 strains in this study were observed, including three changes (L/Q234E, K235 R, and A/S241T) in the puff region (EF loop) of VP2, one substitution (T389A) in the VP3 “knob,” five substitutions (Q798E, V816I, N/D 841E, K/E 849R, and R852K) in VP1, and three substitutions (R1743K, M1767I, and K2061Q) in the 3D region (reference strain RMH001/2013KM576764). The 241T substitution was only found in the HRV-A21 strains in this study but not in any of the three HRV reference sequences. No specific substitutions were found in the BC or DE loops of the VP1 region and other coding gene regions (Fig. 1B).

To assess the genetic variation of the HRV-A21 strains identified in this study, we then conducted a neutralization assay using a HRV-A21 strain isolated in this study and the antiserum against the prototype strain (ATCC VR-1131). The results showed that the antiserum against prototype HRV-A21 could not neutralize the infections of the new isolate, indicating that the mutations in the viral genome represent remarkable antigenic variations.

### Clinical manifestations of HRV-A21-positive patients

We then characterized the manifestation of HRV-A21-positive patients. The signs and symptoms were found to be similar to those of patients who were positive for other HRV genotypes and other respiratory viruses upon hospital admission. The median age of HRV-A21-positive CAP patients were older than that of the five URTI outpatients (70 years vs. 24 years, P = 0.037 by Mann-Whitney U test) (Table 3). Three CAP patients and two URTI patients positive for HRV-A21 had leukocytosis with neutrophilia, and others showed leukopenia at presentation. The viral loads of HRV-A21 in the respiratory samples ranged from 3.7 × 10^4^ to 2.8 × 10^8^ copies/ml, with a median of 2.4 × 10^6^ (IQR 1.3 × 10^6^–2.1 × 10^7^) copies/ml, and no significant differences were observed between the CAP inpatientsand outpatients (1.3 × 10^6^ vs. 1.95 × 10^7^ copies/ml, P = 0.19 by Mann-Whitney U test).

Three of the four HRV-A21-positive CAP patients (coding RMH001, RMH114, and RMH123) deteriorated rapidly after hospitalization and developed several complications, including respiratory failure, septic shock, and acute renal failure. They were then admitted to the intensive care unit and supported with mechanical ventilation. Two of these three patients (coding RMH123 and RMH114) died 26 and 23 days after the onset of illness, respectively. Both fatal cases had underlying diseases; one had cerebrovascular disease and the other had Parkinson’s disease. The other HRV-A21-positive patients recovered.

### Metagenomic analysis of microbial species in HRV-A21-positive patients

**Figure 2:**
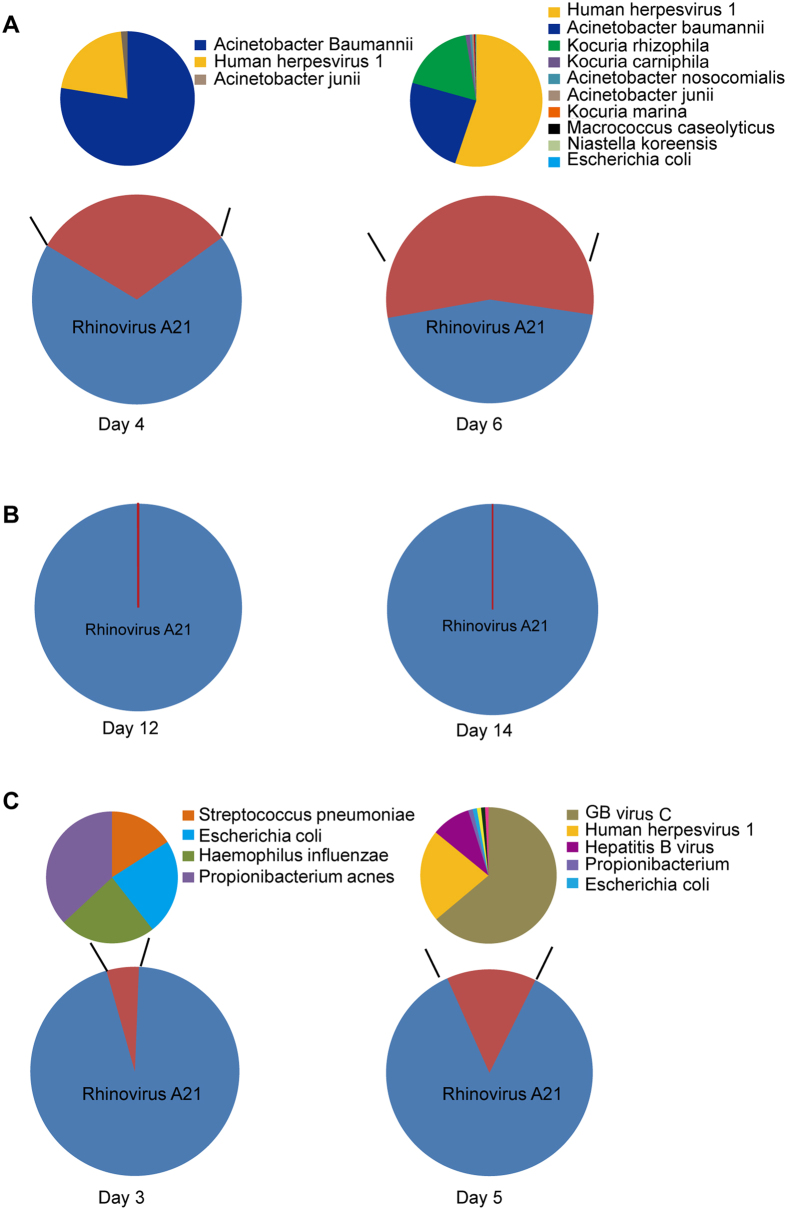
Profiles of the microbial species present in lower respiratory tract samples from patients with severe CAP patients analyzed by deep sequencing. The percentage of microbial species in (**A**) the bronchoalveolar lavage fluid (BALF) taken on days 4 and 6 of patient RMH123 after the onset of symptoms; (**B**) the BALF taken on days 12 and 14 of patient RMH114; and (**C**) the tracheal aspirates taken on days 3 and 5 of patient RMH001, which demonstrates the percentage of rhinovirus reads in most samples.

To determine whether other microbes present as co-pathogens may be related to the observed severity of the HRV-A21 infections (RMH123, RMH114, and RMH001), we carried out metagenomic analysis using the serially collected bronchoalveolar lavage fluid (BALF) or tracheal aspirate samples. HRV-A21 reads were overwhelmingly predominant in the tested samples of the microbial reads, representing 68.7% and 44.8% on days 4 and 9, respectively, in RMH123; 99.9% of the reads on days 12 and 14 in RMH114; and 95.0% and 86.0% of the reads on days 3 and 5 in RMH001 after the onset of symptoms (Fig. 2).

Deep sequencing of the lower respiratory samples from the three severe CAP patients identified 40 single nucleotide polymorphisms (SNPs), including 24 nonsynonymous mutations. These SNPs were located in the VP1, VP2, VP3, 3C, and 3D gene regions (see Supplementary Table S1). Two SNPs switched between minor and major alleles in serial RMH123 viral strains, 3135 (aa 841 in VP1 from E to K), and 5740 (aa 1709 in 3D from C to Y) (reference strain RMH001/2013KM576764). The aa site E841 was the specific site mutation in all the HRV-A21 strains identified in this study. No specific site mutations were found in sequences obtained from RMH123 and RMH144, the two dead cases.

Reads that mapped to known pathogens were also found but at lower level. Sequence data corresponding to *Acinetobacter baumannii* (23.9% and 13.3%) and human herpesvirus (HSV)-1 (6.5% and 0.4%) of the microbial species were detected in RMH123 on days 4 and 6, respectively. In RMH001, on day 3 after the onset of symptoms, reads corresponding to *Escherichia coli, Haemophilus influenza,* and *Streptococcus pneumoniae* accounted for 1.2%, 1.2%, and 0.8% of the identified microbial species, respectively, in BALF. GB virus C, HSV-1, and hepatitis B virus reads were found on day 5 after the onset of symptoms and accounted for 8.8%, 3.0%, and 1.3% of the microbial species, respectively, in the tracheal aspirate.

## Discussion

In this study, we examined the HRV genotypes presented in ARTIs. Most of the identified genotypes were sporadically detected. Unexpectedly, we found that HRV-A21, a clinically rarely reported HRV genotype in literatures before, appears to be present in a range of illnesses from mild URTIs to severe CAP. Moreover, HRV-A21 was the most frequently detected strain among all HRV genotypes detected. HRV-A21 was more frequently detected in patients with severe CAP than in those with non-severe CAP and URTIs. Further analysis showed that the HRV-A21 detected in this study was distinct from the only three strains of HRV previously identified by phylogenic analysis, as demonstrated by the neutralization assay, during which one isolate obtained in this study could not be neutralized by the antiserum against the prototype strain. Metagenomic assay showed that infection with HRV-A21 is strongly associated with sporadic severe CAP in adults.

Respiratory infections with HRV-A21 were scarcely recognized clinically prior to this study. Only three whole HRV-A21 genome sequences were available in GenBank: ATCC VR-1131 (prototype strain), and two sequences obtained directly from clinical patients collected in 2000 and 2010. The only other case of HRV-A21-associated severe respiratory disease was reported in France in 2010, in which one child with pneumonia tested positive for HRV-A21, but only partial VP4/VP2 gene sequences (420 bp) were deposit in GenBank (accession number HE589784)^11^. Our findings on the clinical manifestation and virological features of this genotype provide insights into the pathogenesis of HRVs and new knowledge on the roles of different HRV genotypes in ARTIs, which is informative and necessary to improve the management of ARTIs.

To investigate the association between HRV-A21 and ARTIs, we conducted metagenomics analysis in addition to PCR. For this purpose, we used only lower, not upper, respiratory samples to avoid the interference of mass microbial species, which can be carried in the upper respiratory tract. The data demonstrated that HRV-A21 reads were predominantly present in the BALF or tracheal aspirate samples collected from three patients with severe CAP. Although other agents, including *Acinetobacter baumannii, Escherichia coli, Haemophilus influenza,* and *Streptococcus pneumoniae*, were identified by deep sequencing, they only accounted for a minor part of the microbial reads in early samples, indicating that colonization or possible secondary infections occurred but that they were not the primary causative agents. The HSV-1 reads were identified in the respiratory samples from two patients. However, when compared to those of HRV-A21, they were not the dominant species. It may accounts for the activation of latent viral infections during severe infections. Reads of hepatitis B virus detected in RMH001 in the second respiratory samples might result from the alveolar hemorrhagic effusion. However, the role of these infections in the severity of pneumonia should be intensively investigated in future studies. Taken together, these data suggest that there is an association between HRV-A21 and CAP. In the past, HRV infections were thought to cause changes in the airway microbiome and increase lung inflammation in COPD patients^12^. Whether HRV infection contributes to secondary bacterial infections, such as *Streptococcus pneumoniae*, and whether HRV is a co-pathogen with these agents during the disease process (as is influenza virus) warrants further investigation^13^.

Viral factors and the host immune status are responsible for disease severity during viral infections^13^,^14^. The HRV-A21-positive patients in this study were not immunocompromised, but two of the three severe cases had underlying diseases; thus, the factors that contribute to disease severity require further investigation. Although the number of HRV-A21-positive cases was small, senior age may be a risk factor for the degree of CAP severity in this cohort. Similar to the infections with A/H1N1 2009pdm, H7N9 avian influenza virus, and MERS-CoV, different medical settings are also risk factors for disease severity^15^,^16^,^17^.

Genomic variation may also be a determinant of viral adaption and pathogenesis. In the *Enterovirus* genus, variation can lead to the emergence of new strains or genotypes in epidemics^4^,^18^. For example, the emergent EV-D68 that circulated in the USA in 2014 (http://www.cdc.gov/non-polio-enterovirus/outbreaks/EV-D68-outbreaks.html) contained mutant genomic sequences, which could have been responsible for the outbreak and increased disease severity^4^,^19^. The VP1 BC and DE loops, the VP2 EF loop, and the VP3 knob regions are known to form the major neutralization sites in picornaviruses and the aa mutations in these regions might change the ability to neutralize the virus^20^. The fact that infections of the new HRV A21 isolate could not be neutralized by the antiserum against the prototype strain indicates that the new HRV-A21 strains are antigenic distinct to the prototype strain. Previous studies have also demonstrated that the single aa site mutation in VP1, the VP2 EF loop, and the VP3 knob enhance the virulence of CVB2, B3, and EV71, and the mutation site in the 3D gene, which encodes the RNA-dependent RNA polymerase of EVs, decreased the virulence of EV71 in mice^20^,^21^,^22^. In this study, specific mutations in the VP1, VP2 EF loop, VP3 knob, and 3D regions of HRV-A21 strains were identified by deep sequencing and phylogenetic analysis. However, no specific site mutations were found in sequences obtained from HRV-A21-positive dead cases. The A389 mutation in the VP3 knob of HRV-A21 identified in this study, corresponding to the site in CVB3 has been demonstrated relating to the virulence in CVB3 21. Whether the others HRV-A21 mutations facilitate host adaption and replication, especially in the lower respiratory tract tropism, and result in clinical disease phenotypes remains unclear and warrants further investigation. We also found the switches between minor and major alleles in serially collected HRV strains of RMH123, a dead case, which indicates the occurrence of viral quasispecies during infections. The fact that several HRV variants co-circulate in the population and that we do not have waves of one unique virus indicates a need for precise genotyping of HRV infections. Our findings highlight that HRV should not be underestimated in severe respiratory illness.

Metagenomic analysis has been widely used for viral identification, sequencing, characterization, and origin tracing^23^. The implementation of such methodologies has greatly improved the identification of pathogens, especially for mutant strains and novel viruses. For example, deep sequencing data has been used as direct evidence to identify an H10N8 influenza virus infection in a single human case^24^. Optimization and implementation of the protocols suitable for clinical samples will no doubt improve the microbial diagnosis in clinical practice.

In summary, we identified the predominant infection of HRV-A21 in ARTIs that may be related to life-threatening CAP cases. The findings indicate that there still exists much more to establish regarding the pathogenesis of HRV compared to what we have outlined. Intensive surveillance and precise evaluation of specific HRVs in ARTIs is necessary.

## Methods

### Patients and clinical data

URTI and CAP patients between January 1 and December 31, 2013 were included in this study. URTI patients were recruited by the national respiratory virus surveillance project. The inclusion criteria for URTIs were as follows: at least 18 years of age, acute fever (body temperature ≥38.0 °C), and respiratory symptoms without radiological pulmonary abnormalities. Acute phase sera and nasal and throat swabs were collected from each outpatient when they sought medical care.

Inpatients diagnosed with CAP were recruited through the China Severe Acute Respiratory Infections network (China SARInet). CAP was diagnosed following the guidelines of the Infectious Diseases Society of America and the American Thoracic Society; criteria included age of at least 18 years, with respiratory symptoms, chest roentgenogram, and/or computed tomography confirmed pneumonia^25^. Severe CAP was diagnosed according to published criteria^26^. Pneumonia patients with confirmed non-inflammatory pulmonary disease were excluded. BALF or tracheal aspirate specimens and sera were collected from each inpatient when clinically indicated. BALF or tracheal aspirate specimens were collected as second respiratory samples in patients with mechanical ventilation, followed by serial sputum collection. The clinical information for each enrolled patient was recorded using a standard form.

The study was approved by the Medical Ethics Review Board of the Institute of Pathogen Biology, Chinese Academy of Medical Sciences. All the methods were carried out in accordance with relevant guidelines and regulations, including any relevant details. Written informed consent for scientific assessment was obtained from each patient as part of the clinical treatment contract.

### Molecular detection of HRVs

Total nucleic acids were extracted from respiratory and blood samples using a NucliSens easyMAG apparatus (bioMerieux, Marcy l′Etoile, France). The presence of HRVs and other common respiratory pathogens were screened using the fast-track diagnostic respiratory pathogens 21 plus kit (2013 version, Fast-track diagnostics, Junglinster, Luxembourg), which included HRVs, IFVA and B, human coronaviruses (HCoV, OC43, 229E, NL63, and HKU1), parainfluenza viruses, human metapneumovirus (A and B), EV, RSV (A and B), adenovirus, bocavirus, parechovirus, *Mycoplasma pneumoniae, Chlamydia pneumoniae, Streptococcus pneumoniae, Haemophilus influenzae B,* and *Staphylococcus aureus*. Bacterial culture was performed on lower respiratory samples and acute sera using an ATBExpression automatic bacterial identification instrument (bioMerieux, Marcy l′Etoile, France).

### HRV genotyping, load quantification, and phylogenic analysis

Conventional reverse transcriptase PCR (RT-PCR) targeting the 5′-untranslated region (UTR)/viral protein (VP) gene 4 and the VP4/VP2 region was used for HRV screening and genotyping as described elsewhere^27^,^28^,^29^. Briefly, the PCR products were sequenced directly using an ABI 3730xl automatic DNA analyzer (Life Technologies, Grand Island, NY, USA). Genotypes were determined by phylogenic analysis using the amplified gene sequences compared to the reference HRV genotype sequence deposited in GenBank and by MEGA 5.1 using the neighbor-joining method with Kimura’s two-parameter model and 1,000 bootstrap pseudo-replicates^30^. The HRV viral loads in the respiratory samples were determined using an HRV 5′-UTR quantitative real-time RT-PCR assay and standard curve analysis. The standard RNA transcript was amplified *in vitro* from the plasmid containing gene fragments using primers targeting UTR/VP4 29.

### Metagenomic analysis

The lower respiratory samples collected from severe CAP patients were used to construct the cDNA libraries and sequenced using an Ion Torrent platform with a type 318 chip (Thermo Fisher Scientific Inc, USA). The samples were treated with Turbo DNase (Life Technologies, USA) and RNAse One (Promega, USA) to decrease the host genome background according to the manufacturer’s instructions. Total nucleic acids were extracted and amplified using the Ovation RNA-Seq System (Nugen, California, USA). The libraries for deep sequencing were constructed using the NEBNext Fast DNA Library Prep set for the Ion Torrent (New England Biolabs). Alignments with mapping quality scores less than 30 and reads containing fewer than 50 nucleotides were excluded from the following analysis. The taxonomic assignment was performed using MEGAN software (version 5.2.3) with the parameters “Min Support 50, Min score 80, Top percent 10” 31. All reads assigned to the *Enterovirus* genus were retrieved and aligned to the consensus sequence of the reference strain, HRV-A21_p1177_sR3307_2010 (GenBank accession number JN837693), using BWA (version 0.7.5, BWA-MEM algorithm) and GATK (version 2.5.2)^32^,^33^. Pileup files were generated by SAMtools, and the allele frequency was calculated as the proportion of reads presenting the allele amongst all reads mapped to this position^34^.

### HRV-A21 genome amplification

Primers targeting the complete genome of HRV-A21 were designed according to the HRV-A21 sequences obtained from deep sequencing and used to amplify genomic sequences from other HRV-A21-positive samples (see Supplementary Table S2). The 5′ and 3′ UTR sequences were determined via the RACE System (Invitrogen, Carlsbad, CA, USA) according to the manufacturer’s protocol. Total RNA from respiratory specimens was converted to cDNA using combined random primers, oligo(dT) primers, and the SuperScript III reverse transcription system (Invitrogen, Carlsbad, CA). PCR was performed using the following conditions: 94 °C for 5 min, 40 cycles of amplification at 94 °C for 30 s, 50 °C for 45 s, and 72 °C for 90 s, with a terminal elongation step at 72 °C for 10 min. PCR products were sequenced directly and assembled manually through alignment to the reference strain (GenBank accession number KM576764). The whole genome and viral protein genes were used to construct phylogenetic trees in MEGA 5.1 using the neighbor-joining method with Kimura’s two-parameter model and 1,000 bootstrap pseudo-replicates^30^.

### Virus isolation and neutralization assay

The HRV-A21 virus was isolated at 33 °C using a human cervical carcinoma cell line (H1-Hela; ATCC CRL-1958) from BALF samples of patient RMH001 collected on day 3 after the onset of symptoms. Cytopathic effects could be visualized. A neutralization assay was then carried out by using the reference antiserum against the prototype HRV-A21 strain (ATCC VR-1131 AS/GP) and the HRV-A21 isolate in accordance with the World Health Organization (WHO) standard procedures for poliovirus^35^.

### Nucleotide sequence accession numbers

The partial sequences obtained from genotyping were deposited in GenBank under the accession numbers: KR871703, KR871703, KR871714, KT229617, KT229619, KT229620, KU672654, KU859961, KU859962, KR873691 to KR873699, KR871693 to KR871699, KX384582 to KX384588, and KR871706 to KR871709. The GenBank accession numbers of the full HRV-A21 genomes obtained are KM576764 to KM576767, KR871674 to KR871677, and KR090587. The project number of the deep sequencing data is PRJNA335944.

### Statistical analyses

Pearson’s Chi square tests or Fisher’s exact tests were used for categorical variables and Mann-Whitney U tests was used to compare continuous variables by using R version 2.15.3 (R Core Team. R: A language and environment for statistical computing. R Foundation for Statistical Computing, Vienna, Austria). *P* < 0.05 was considered statistically significant.

## Additional Information

**How to cite this article**: Ren, L. *et al*. Genotyping of human rhinovirus in adult patients with acute respiratory infections identified predominant infections of genotype A21. *Sci. Rep.* **7**, 41601; doi: 10.1038/srep41601 (2017).

**Publisher's note:** Springer Nature remains neutral with regard to jurisdictional claims in published maps and institutional affiliations.

## Supplementary Material

Supplementary Tables

## Figures and Tables

**Table 1:** Human rhinovirus genotypes detected in patients with respiratory infections.

	Severe CAP	Non-severe CAP	URTIs	Statistical analysis^§^
No. tested	38	109	291	
Negative^a^	19 (50.0)^b^	58 (53.2)	192 (66.0)	P = 0.02
Positive	19 (50.0)	51 (46.8)	99 (34.0)	
Frequency of HRV				
HRV	9 (23.7)	6 (5.5)	27 (9.3)	P = 0.01
HRV-A	6 (15.8)	5 (4.6)	24 (8.2)	P = 0.09
HRV-A21	3 (7.9)	1 (0.9)	5 (1.7)	P = 0.047
Other genotypes	A2, A13, A33	A9, A53, A68 (2)	A2, A28, A53, A58, A60, A61(3), A68, A96, A98 (2), untyped (7)	
HRV-B	2 (5.3)	1 (1.8)	2 (0.7)	P = 0.095
	B35, B48	B84	B27, B84	
HRV-C	1 (2.6)	0	1 (0.3)	P = 0.228

^a^The positive and negative results were determined according to the results of PCR assays using an FTD respiratory pathogens 21 plus kit; ^b^Numbers in parentheses indicate the percentages of the number of times the virus was detected relative to the total number of samples; ^c^The P values are from Fisher’s exact test; CAP, community-acquired pneumonia; URTIs, upper respiratory tract infections; NA, not available.

**Table 2:** Nucleotide and amino acid identities compared to previously identified HRV-A21 strains^a^.

Identity (nt/aa)%												
Gene region	5′-UTR	VP4	VP2	VP3	VP1	2A	2B	2C	3A	3B	3C	3D
JQ747747	97.2	94.2/100.0	92.5/96.1	91.4/96.6	92.1/96.1	95.3/100.0	93.3/100.0	92.7/99·0	93.5/98.7	90.4/100.0	91.6/99.4	92.1/98.2
JN837693	96.1	92.2/100.0	91.0/96.5	91.2/96.2	91.6/96.1	91.5/100.0	93.3/98.9	92.0/98.7	90.9/98.7	90.4/100.0	91.8/100.0	92.1/98.2
FJ445121	98.6	92.7/98.5	88.8/94.6	90.8/95.4	90.4/95.1	90.8/99.2	94.0/100.0	92.1/98.7	90.0/97.4	88.8/100.0	90.5/97.8	91.3/97.9

^a^The strain of RMH001/2013 (accession number, KM576764) was used as representative sequence.

**Table 3:** Clinical features of patients positive for human rhinovirus A21.

Code	Age (ys)	Gender	Diagnosis	Date of Onset of Symptoms	Tm^a^ (°C)	Signs and symptoms upon admission	Clinic laboratory test		Sampling date	Samples	Viral titer (copies/ml)
							White blood × 10^9^/L	Neutrophile granulocyte%			
RMH123	72	M	Severe CAP	26 Feb 2013	39.2	Fever, cough, sputum, sore throat, dyspnea, moist rales	16.91	97.2	01 Mar 201303 Mar2013	BALFSputum	1.8 × 10^6^
RMH114	73	M	Severe CAP	9 Apr 2013	38	Fever, cough, sputum, sore throat, dyspnea, moist rales	19.58	92.8	20 Apr 201322 Apr 2013	BALFBALF	1.8 × 10^7^
RMH001	35	M	Severe CAP	6 Apr 2013	38.6	Fever, cough, dyspnea	8.6	95.5	08 Apr 201310 Apr 2013	Tracheal aspirateTracheal aspirate	2.8 × 10^8^
RMH97	68	F	CAP	12 Jul 2013	40	Fever, cough, sputum	19.72	91.3	23 Jul 2013	BALF	2.1 × 10^7^
PUMCH13351	21	F	URTI	19 Feb 2013	38.5	Fever, cough, sputum, sore throat, runny nose	15	72.9	26 Feb 2013	Nasal and throat swabs, serum	1.3 × 10^6^
PUMCH13364	27	M	URTI	1 Apr 2013	38.4	Fever, cough, sputum, sore throat, headache, runny nose	9.05	80.5	2 Apr 2013	Nasal and throat swabs	2.4 × 10^6^
PUMCH13371	39	M	URTI	4 Apr 2013	38	Fever, sore throat, headache, runny nose, chilly, feeble	9.34	80	5 Apr 2013	Nasal and throat swabs	3.1 × 10^7^
PUMCH13478	21	F	URTI	16 Jun 2013	38	Fever, cough, sputum, sore throat, headache, runny nose, chilly, vomit	8.89	84.3	17 Jun 2013	Nasal and throat swabs	3.7 × 10^4^
PUMCH13515	24	M	URTIs	27 Sep 2013	38.5	Fever, sore throat, runny nose	12.64	76.2	29 Sep 2013	Nasal and throat swabs	1.0 × 10^6^

^a^Tm, the highest body temperature before admission; Ys, years; URTI, upper respiratory tract infection; BALF, bronchoalveolar lavage fluid.
