# Supplementary material for: Genotyping of human rhinovirus in adult patients with acute respiratory infections identified predominant infections of genotype A21
Source: Sci Rep. 2017 Jan 27;7:41601. doi: 10.1038/srep41601 (PMC5269714; doi:10.1038/srep41601)
Supplement: Supplementary Tables [file srep41601-s1.doc]

**Genotyping of human rhinovirus in adult patients with acute respiratory infections identified predominant infections of genotype A21**

Lili Ren^1 #^, Donghong Yang^2 #^, Xianwen Ren^3 #^, Mingkun Li^4^, Xinlin Mu^2^, Qi Wang^5^, Jie Cao^6^, Ke Hu^7^, Chunliang Yan^8^, Hongwei Fan^9^, Xiangxin Li^10^, Yusheng Chen^11^, Ruiqin Wang^12^, Fucheng An^13^, Shuchang An^12^, Ming Luo^14^, Ying Wang^1^, Yan Xiao^1^, Zichun Xiang^1^, Yan Xiao^15^, Li Li^15^, Fang Huang^14^, Qi Jin^3^†, Zhancheng Gao^2^†, Jianwei Wang^1^†.

^1^MOH Key Laboratory of Systems Biology of Pathogens and Christophe Mérieux Laboratory, IPB, CAMS-Fondation Mérieux, Institute of Pathogen Biology (IPB), Chinese Academy of Medical Sciences (CAMS) & Peking Union Medical College, Beijing 100730, P. R. China

^2^Department of Respiratory and Critical Care Medicine, Peking University People’s Hospital, Beijing100044, P. R. China

^3^MOH Key Laboratory of Systems Biology of Pathogens, Institute of Pathogen Biology, Chinese Academy of Medical Sciences & Peking Union Medical College, Beijing 100176, P. R. China

^4^Fondation Mérieux, Lyon 69365, France

^5^Department of Respiratory Medicine, The Second Affiliated Hospital of Dalian Medical University, Dalian 116027, P. R. China

^6^Department of Respiratory Medicine, Tianjin Medical University General Hospital, Tianjin 300052, P. R. China

^7^Department of Respiratory Medicine, Renmin Hospital of Wuhan University, Wuhan 430060, P. R. China

^8^Department of Respiratory & Critical Care Medicine, Beijing Aerospace General Hospital, Beijing 100076, P. R. China

^9^Peking Union Medical College Hospital, Chinese Academy of Medical Sciences & Peking Union Medical College, Beijing 100730, P. R. China

^10^Department of Respiratory Medicine, Beijing Changping Hospital, Beijing 102200, P. R. China

^11^Department of Respiratory Medicine, Fujian Provincial Hospital, Fuzhou 350001, P. R. China

^12^Department of Respiratory Medicine, The First Affiliated Hospital of Tsinghua University, Beijing 100016, P. R. China

^13^Department of Respiratory Medicine, Mentougou District Hospital, Beijing 102300, P. R. China

^14^Beijing Center for Disease Prevention and Control. No.16, Hepingli Middle Avenue of Dongcheng district, Beijing 100013, P. R. China

^15^Institute of Pathogen Biology, Chinese Academy of Medical Sciences & Peking Union Medical College, Beijing 100176, P. R. China

^#^Contributed equally

†Also contributed equally

**Correspondence to:**

Dr. Jianwei Wang

No.9 Dong Dan San Tiao, Dongcheng District

Beijing 100730, P. R. China

Tel/Fax: 86-10-67828516

E-mail: [wangjw28@163.com](mailto:wangjw28@163.com)

Zhancheng Gao, MD, PhD

No. 11 Xizhimen South Street, Xicheng District

Peking University People’s Hospital, Beijing 100044, P. R. China

Tel/Fax: 86-10-88324886/68318386

E-mail: zcgao@bjmu.edu.cn

Dr. Qi Jin

No.6 Rong Jing Dong Jie, Daxing District

Beijing 100176, P. R. China

Tel/Fax: 86-10-67877732

E-mail:  [zdsys@vip.sina.com](mailto:wangjw28@163.com)

**Table S1. Single nucleotide polymorphisms of the HRV-A21 sequences obtained by deep sequencing from serially collected lower respiratory samples of severe pneumonia patients.**

| Gene region | Site^a^ | Code | Days after onset of symptoms | Major allele | Amino acids | Frequency of major allele | Minor allele | Frequency of minor allele^b^ |
| --- | --- | --- | --- | --- | --- | --- | --- | --- |
| 5'-UTR | 348 | RMH001 | 3 | C |  | 1 |  |  |
|  |  | RMH001 | 5 | C |  | 1 |  |  |
|  |  | RMH123 | 4 | T |  | 0.979 |  | 0.021 |
|  |  | RMH123 | 6 | T |  | 1 |  |  |
|  |  | RMH114 | 12 | T |  | 1 |  |  |
|  |  | RMH114 | 14 | T |  | 1 |  |  |
|  | 514 | RMH001 | 3 | T |  | 1 |  |  |
|  |  | RMH001 | 5 | T |  | 1 |  |  |
|  |  | RMH123 | 4 | C |  | 1 |  |  |
|  |  | RMH123 | 6 | C |  | 1 |  |  |
|  |  | RMH114 | 12 | T |  | 1 |  |  |
|  |  | RMH114 | 14 | T |  | 1 |  |  |
|  | 568 | RMH001 | 3 | T |  | 1 |  |  |
|  |  | RMH001 | 5 | T |  | 1 |  |  |
|  |  | RMH123 | 4 | T |  | 0.955 |  | 0.038 |
|  |  | RMH123 | 6 | T |  | 0.968 |  | 0.029 |
|  |  | RMH114 | 12 | C |  | 1 |  |  |
|  |  | RMH114 | 14 | C |  | 1 |  |  |
| VP4 | 680 | RMH001 | 3 | T | G | 1 |  |  |
|  |  | RMH001 | 5 | T | G | 1 |  |  |
|  |  | RMH123 | 4 | T | G | 1 |  |  |
|  |  | RMH123 | 6 | T | G | 1 |  |  |
|  |  | RMH114 | 12 | C | G | 1 |  |  |
|  |  | RMH114 | 14 | C | G | 1 |  |  |
|  | 713 | RMH001 | 3 | C | F | 1 |  |  |
|  |  | RMH001 | 5 | C | F | 1 |  |  |
|  |  | RMH123 | 4 | T | F | 0.918 | A | 0.049 |
|  |  | RMH123 | 6 | T | F | 0.927 | A | 0.063 |
|  |  | RMH114 | 12 | C | F | 1 |  |  |
|  |  | RMH114 | 14 | C | F | 1 |  |  |
|  | 722 | RMH001 | 3 | T | A | 1 |  |  |
|  |  | RMH001 | 5 | T | A | 1 |  |  |
|  |  | RMH123 | 4 | C | A | 1 |  |  |
|  |  | RMH123 | 6 | C | A | 1 |  |  |
|  |  | RMH114 | 12 | C | A | 1 |  |  |
|  |  | RMH114 | 14 | C | A | 1 |  |  |
|  | 773 | RMH001 | 3 | T | F | 1 |  |  |
|  |  | RMH001 | 5 | T | F | 1 |  |  |
|  |  | RMH123 | 4 | C | F | 1 |  |  |
|  |  | RMH123 | 6 | C | F | 1 |  |  |
|  |  | RMH114 | 12 | T | F | 1 |  |  |
|  |  | RMH114 | 14 | T | F | 1 |  |  |
| VP2 | 1016 | RMH001 | 3 | G | T | 1 |  |  |
|  |  | RMH001 | 5 | G | T | 1 |  |  |
|  |  | RMH123 | 4 | A | T | 1 |  |  |
|  |  | RMH123 | 6 | A | T | 1 |  |  |
|  |  | RMH114 | 12 | A | T | 1 |  |  |
|  |  | RMH114 | 14 | A | T | 1 |  |  |
|  | 1286 | RMH001 | 3 | C | D | 1 |  |  |
|  |  | RMH001 | 5 | C | D | 1 |  |  |
|  |  | RMH123 | 4 | C | D | 1 |  |  |
|  |  | RMH123 | 6 | C | D | 1 |  |  |
|  |  | RMH114 | 12 | T | D | 1 |  |  |
|  |  | RMH114 | 14 | T | D | 1 |  |  |
|  | 1412 | RMH001 | 3 | T | N | 1 |  |  |
|  |  | RMH001 | 5 | T | N | 1 |  |  |
|  |  | RMH123 | 4 | T | N | 1 |  |  |
|  |  | RMH123 | 6 | T | N | 1 |  |  |
|  |  | RMH114 | 12 | C | N | 1 |  |  |
|  |  | RMH114 | 14 | C | N | 1 |  |  |
|  | 1433 | RMH001 | 3 | T | V | 1 |  |  |
|  |  | RMH001 | 5 | T | V | 1 |  |  |
|  |  | RMH123 | 4 | T | V | 1 |  |  |
|  |  | RMH123 | 6 | T | V | 1 |  |  |
|  |  | RMH114 | 12 | C | V | 1 |  |  |
|  |  | RMH114 | 14 | C | V | 1 |  |  |
|  | 1530 | RMH001 | 3 | A | T | 1 |  |  |
|  |  | RMH001 | 5 | A | T | 1 |  |  |
|  |  | RMH123 | 4 | T | S | 1 |  |  |
|  |  | RMH123 | 6 | T | S | 1 |  |  |
|  |  | RMH114 | 12 | A | T | 1 |  |  |
|  |  | RMH114 | 14 | A | T | 1 |  |  |
| VP3 | 1781 | RMH001 | 3 | C | A | 1 |  |  |
|  |  | RMH001 | 5 | C | A | 1 |  |  |
|  |  | RMH123 | 4 | C | A | 1 |  |  |
|  |  | RMH123 | 6 | C | A | 1 |  |  |
|  |  | RMH114 | 12 | A | A | 1 |  |  |
|  |  | RMH114 | 14 | A | A | 1 |  |  |
|  | 1822 | RMH001 | 3 | A | N | 1 |  |  |
|  |  | RMH001 | 5 | A | N | 1 |  |  |
|  |  | RMH123 | 4 | T | I | 1 |  |  |
|  |  | RMH123 | 6 | T | I | 1 |  |  |
|  |  | RMH114 | 12 | A | N | 1 |  |  |
|  |  | RMH114 | 14 | A | N | 1 |  |  |
|  | 1833 | RMH001 | 3 | A | N | 1 |  |  |
|  |  | RMH001 | 5 | A | N | 1 |  |  |
|  |  | RMH123 | 4 | G | D | 0.748 | A | 0.251 |
|  |  | RMH123 | 6 | G | D | 0.858 | A | 0.142 |
|  |  | RMH114 | 12 | A | N | 1 |  |  |
|  |  | RMH114 | 14 | A | N | 1 |  |  |
|  | 2055 | RMH001 | 3 | T | L | 1 |  |  |
|  |  | RMH001 | 5 | T | L | 1 |  |  |
|  |  | RMH123 | 4 | C | L | 1 |  |  |
|  |  | RMH123 | 6 | C | L | 1 |  |  |
|  |  | RMH114 | 12 | T | L | 1 |  |  |
|  |  | RMH114 | 14 | T | L | 1 |  |  |
|  | 2252 | RMH001 | 3 | C | S | 1 |  |  |
|  |  | RMH001 | 5 | C | S | 1 |  |  |
|  |  | RMH123 | 4 | C | S | 1 |  |  |
|  |  | RMH123 | 6 | C | S | 1 |  |  |
|  |  | RMH114 | 12 | T | S | 1 |  |  |
|  |  | RMH114 | 14 | T | S | 1 |  |  |
|  | 2276 | RMH001 | 3 | G | R | 1 |  |  |
|  |  | RMH001 | 5 | G | R | 1 |  |  |
|  |  | RMH123 | 4 | A | R | 1 |  |  |
|  |  | RMH123 | 6 | A | R | 1 |  |  |
|  |  | RMH114 | 12 | G | R | 1 |  |  |
|  |  | RMH114 | 14 | G | R | 0.967 | +A | 0.032 |
|  | 2288 | RMH001 | 3 | C | D | 1 |  |  |
|  |  | RMH001 | 5 | C | D | 1 |  |  |
|  |  | RMH123 | 4 | T | D | 1 |  |  |
|  |  | RMH123 | 6 | T | D | 1 |  |  |
|  |  | RMH114 | 12 | C | D | 1 |  |  |
|  |  | RMH114 | 14 | C | D | 1 |  |  |
| VP1 | 2906 | RMH001 | 3 | C | Y | 1 |  |  |
|  |  | RMH001 | 5 | C | Y | 1 |  |  |
|  |  | RMH123 | 4 | C | Y | 1 |  |  |
|  |  | RMH123 | 6 | C | Y | 1 |  |  |
|  |  | RMH114 | 12 | T | Y | 1 |  |  |
|  |  | RMH114 | 14 | T | Y | 1 |  |  |
|  | 2940 | RMH001 | 3 | G | D | 1 |  |  |
|  |  | RMH001 | 5 | G | D | 1 |  |  |
|  |  | RMH123 | 4 | A | N | 1 |  |  |
|  |  | RMH123 | 6 | A | N | 1 |  |  |
|  |  | RMH114 | 12 | G | D | 1 |  |  |
|  |  | RMH114 | 14 | G | D | 1 |  |  |
|  | 3015 | RMH001 | 3 | C | H | 1 |  |  |
|  |  | RMH001 | 5 | C | H | 1 |  |  |
|  |  | RMH123 | 4 | A | N | 0.897 | - | 0.086 |
|  |  | RMH123 | 6 | A | N | 0.913 | - | 0.083 |
|  |  | RMH114 | 12 | C | H | 1 |  |  |
|  |  | RMH114 | 14 | C | H | 1 |  |  |
|  | 3080 | RMH001 | 3 | A | R | 1 |  |  |
|  |  | RMH001 | 5 | A | R | 1 |  |  |
|  |  | RMH123 | 4 | A | R | 1 |  |  |
|  |  | RMH123 | 6 | A | R | 1 |  |  |
|  |  | RMH114 | 12 | G | R | 1 |  |  |
|  |  | RMH114 | 14 | G | R | 1 |  |  |
|  | 3111 | RMH001 | 3 | T | Y | 1 |  |  |
|  |  | RMH001 | 5 | T | Y | 1 |  |  |
|  |  | RMH123 | 4 | C | H | 0.939 | +A | 0.059 |
|  |  | RMH123 | 6 | C | H | 0.972 | +A | 0.027 |
|  |  | RMH114 | 12 | C | H | 0.943 | - | 0.037 |
|  |  | RMH114 | 14 | C | H | 0.948 | - | 0.033 |
|  | 3135 | RMH001 | 3 | G | E | 1 |  |  |
|  |  | RMH001 | 5 | G | E | 0.976 | +A | 0.024 |
|  |  | RMH123 | 4 | ***G*** | ***E*** | 0.705 | ***A*** | 0.268 |
|  |  | RMH123 | 6 | ***A*** | ***K*** | 0.871 | ***G*** | 0.071 |
|  |  | RMH114 | 12 | G | E | 0.977 | +A | 0.022 |
|  |  | RMH114 | 14 | G | E | 0.977 | +A | 0.022 |
| 2A | 3344 | RMH001 | 3 | C | D | 1 |  |  |
|  |  | RMH001 | 5 | C | D | 1 |  |  |
|  |  | RMH123 | 4 | T | D | 1 |  |  |
|  |  | RMH123 | 6 | T | D | 1 |  |  |
|  |  | RMH114 | 12 | C | D | 1 |  |  |
|  |  | RMH114 | 14 | C | D | 1 |  |  |
|  | 3536 | RMH001 | 3 | T | I | 1 |  |  |
|  |  | RMH001 | 5 | T | I | 1 |  |  |
|  |  | RMH123 | 4 | C | I | 1 |  |  |
|  |  | RMH123 | 6 | C | I | 1 |  |  |
|  |  | RMH114 | 12 | T | I | 1 |  |  |
|  |  | RMH114 | 14 | T | I | 1 |  |  |
| 2B | 3677 | RMH001 | 3 | G | K | 1 |  |  |
|  |  | RMH001 | 5 | G | K | 1 |  |  |
|  |  | RMH123 | 4 | G | K | 0.936 | A | 0.063 |
|  |  | RMH123 | 6 | G | K | 0.938 | A | 0.061 |
|  |  | RMH114 | 12 | A | K | 1 |  |  |
|  |  | RMH114 | 14 | A | K | 1 |  |  |
|  | 3824 | RMH001 | 3 | T | C | 1 |  |  |
|  |  | RMH001 | 5 | T | C | 1 |  |  |
|  |  | RMH123 | 4 | C | C | 0.967 | T | 0.031 |
|  |  | RMH123 | 6 | C | C | 1 |  |  |
|  |  | RMH114 | 12 | T | C | 1 |  |  |
|  |  | RMH114 | 14 | T | C | 1 |  |  |
| 2C | 4133 | RMH001 | 3 | G | T | 1 |  |  |
|  |  | RMH001 | 5 | G | T | 1 |  |  |
|  |  | RMH123 | 4 | G | T | 1 |  |  |
|  |  | RMH123 | 6 | G | T | 1 |  |  |
|  |  | RMH114 | 12 | A | T | 1 |  |  |
|  |  | RMH114 | 14 | A | T | 1 |  |  |
|  | 4268 | RMH001 | 3 | T | G | 1 |  |  |
|  |  | RMH001 | 5 | T | G | 1 |  |  |
|  |  | RMH123 | 4 | T | G | 1 |  |  |
|  |  | RMH123 | 6 | T | G | 1 |  |  |
|  |  | RMH114 | 12 | C | G | 1 |  |  |
|  |  | RMH114 | 14 | C | G | 1 |  |  |
|  | 4532 | RMH001 | 3 | G | L | 1 |  |  |
|  |  | RMH001 | 5 | G | L | 1 |  |  |
|  |  | RMH123 | 4 | G | L | 1 |  |  |
|  |  | RMH123 | 6 | G | L | 1 |  |  |
|  |  | RMH114 | 12 | A | L | 1 |  |  |
|  |  | RMH114 | 14 | A | L | 1 |  |  |
| 3A | 4952 | RMH001 | 3 | A | E | 1 |  |  |
|  |  | RMH001 | 5 | A | E | 1 |  |  |
|  |  | RMH123 | 4 | A | E | 1 |  |  |
|  |  | RMH123 | 6 | A | E | 1 |  |  |
|  |  | RMH114 | 12 | G | E | 1 |  |  |
|  |  | RMH114 | 14 | G | E | 1 |  |  |
| 3C | 5276 | RMH001 | 3 | C | H | 1 |  |  |
|  |  | RMH001 | 5 | C | H | 1 |  |  |
|  |  | RMH123 | 4 | C | H | 1 |  |  |
|  |  | RMH123 | 6 | C | H | 1 |  |  |
|  |  | RMH114 | 12 | T | H | 1 |  |  |
|  |  | RMH114 | 14 | T | H | 1 |  |  |
|  | 5330 | RMH001 | 3 | T | D | 1 |  |  |
|  |  | RMH001 | 5 | T | D | 1 |  |  |
|  |  | RMH123 | 4 | C | D | 0.949 | +A | 0.052 |
|  |  | RMH123 | 6 | C | D | 0.948 | +A | 0.053 |
|  |  | RMH114 | 12 | C | D | 0.945 | +A | 0.057 |
|  |  | RMH114 | 14 | C | D | 0.95 | +A | 0.051 |
|  | 5545 | RMH001 | 3 | C | T | 1 |  |  |
|  |  | RMH001 | 5 | C | T | 1 |  |  |
|  |  | RMH123 | 4 | C | T | 0.892 | - | 0.063 |
|  |  | RMH123 | 6 | C | T | 0.957 | - | 0.028 |
|  |  | RMH114 | 12 | T | I | 1 |  |  |
|  |  | RMH114 | 14 | T | I | 1 |  |  |
| 3D | 5740 | RMH001 | 3 | G | C | 1 |  |  |
|  |  | RMH001 | 5 | G | C | 1 |  |  |
|  |  | RMH123 | 4 | ***G*** | ***C*** | 0.801 | ***A*** | 0.198 |
|  |  | RMH123 | 6 | ***A*** | ***Y*** | 0.617 | ***G*** | 0.382 |
|  |  | RMH114 | 12 | G | C | 1 |  |  |
|  |  | RMH114 | 14 | G | C | 1 |  |  |
|  | 5777 | RMH001 | 3 | A | K | 1 |  |  |
|  |  | RMH001 | 5 | A | K | 1 |  |  |
|  |  | RMH123 | 4 | G | K | 1 |  |  |
|  |  | RMH123 | 6 | G | K | 1 |  |  |
|  |  | RMH114 | 12 | G | K | 1 |  |  |
|  |  | RMH114 | 14 | G | K | 1 |  |  |
|  | 6098 | RMH001 | 3 | T | I | 1 |  |  |
|  |  | RMH001 | 5 | T | I | 1 |  |  |
|  |  | RMH123 | 4 | C | I | 1 |  |  |
|  |  | RMH123 | 6 | C | I | 1 |  |  |
|  |  | RMH114 | 12 | C | I | 1 |  |  |
|  |  | RMH114 | 14 | C | I | 1 |  |  |
|  | 6200 | RMH001 | 3 | A | K | 1 |  |  |
|  |  | RMH001 | 5 | A | K | 1 |  |  |
|  |  | RMH123 | 4 | G | K | 0.960 | A | 0.040 |
|  |  | RMH123 | 6 | G | K | 0.961 | A | 0.039 |
|  |  | RMH114 | 12 | A | K | 1 |  |  |
|  |  | RMH114 | 14 | A | K | 1 |  |  |
| ^a^The strain of RMH001/2013 (accession number, KM576764) was used as representative sequence. ^b^Minor allele with frequency <2% or supported by less than five reads were removed from the table. | | | | | | | | |

**Table S2. Primers used for the amplification of HRV-A21 whole genome sequence.**

| **Code^a^** | **Primer sequence** |
| --- | --- |
| HRV21-1F | TAAAACTGGATCCAGGTTGTTCCCA |
| HRV21-846R | AACCACATGCCTCAACAGTTGGAGA |
| HRV21-114F | AGGTTATCAATACGACCAATAGGTG |
| HRV21-964R | GTGGCATCTTGTGGTGTCAAGT |
| HRV21-830F | TGTTGAGGCATGTGGTTATTCAGAT |
| HRV21-1730R | TAAATTTGTAACCTGCCCTGGGA |
| HRV21-1648F | CAGATGACTTTCAATCTCCAAGTGC |
| HRV21-2554R | ATGCACCCTGATCTACCCAGAAA |
| HRV21-2430F | GCCGCAGAAACTGGACACAC |
| HRV21-3488R | ACATGGCCCCTCGCCTATTA |
| HRV21-3388F | TCCCAATTAAAGTCACTAGCCATG |
| HRV21-4485R | CCATCGGGGGTATAAAAGTAACAC |
| HRV21-4350F | CCTGACCCTAAATACTTTGATGGAT |
| HRV21-5460R | AATTGCAATCTGGGTAGTCATCTTC |
| HRV21-5285F | AGGGAAGGAAGTTCAAATTGATGG |
| HRV21-6313R | CCTGGGTTCAAGTGAAATTTAGAGA |
| HRV21-6202F | AAAAGATTTCTGCCGGGAAGAC |
| HRV21-6852R | ACCAGCACCAACACTGCGTA |
| HRV21-7077R | CATACCATTCATGCAGAAGCAGATC |
| HRV21-VP1F | AATCCTGTAGAAAATTACATAGATG |
| HRV21-VP1R | AACTGTGAAAATTGTTTTTCTGGATC |
| HRV21-3 race F | CATCCAACATTTCCAATTGAGGAG |
| HRV21-5 race R1 | CGCCAGTGGGGGATTTCTAGCCTCA |
| HRV21-5 race R2 | TAGCCTCATCTGCCAGGTCTACTAT |

^a^ Reference sequence RMH001/2013, GenBank accession number KM576764.
